# Circulating bile acid profile characteristics in PCOS patients and the role of bile acids in predicting the pathogenesis of PCOS

**DOI:** 10.3389/fendo.2023.1239276

**Published:** 2023-08-24

**Authors:** Jie Yu, Yi Zhang, Yuchen Zhu, Yushan Li, Siyu Lin, Wei Liu, Tao Tao

**Affiliations:** Department of Endocrinology and Metabolism, Renji Hospital, School of Medicine, Shanghai Jiao Tong University, Shanghai, China

**Keywords:** bile acid, OPLS-DA model, PCOS, biomarker, HPLC/MS

## Abstract

**Background:**

The metabolic profile of bile acids and their potential role as biomarkers in the pathogenesis of polycystic ovary syndrome (PCOS) have not been thoroughly characterized. Assessing their predictive value for PCOS is of significant importance.

**Methods:**

In this study, we enrolled 408 women with PCOS and 204 non-PCOS controls. The serum bile acid profile was measured using high-performance liquid chromatography-tandem mass spectrometry (LC/MS). We analyzed the differences in serum bile acid profiles between PCOS patients using the OPLS-DA model. Additionally, we examined the relationship between bile acid profiles and parameters related to glucose metabolism and hyperandrogenism. ROC analysis was employed to identify potential biomarkers for PCOS pathogenesis. XGboost was utilized for cross-validation.

**Results:**

The bile acid profile was found to be altered in PCOS patients. Specifically, the primary and secondary unconjugated bile acid fractions were significantly higher in the PCOS population. We identified five bile acid metabolite candidates that exhibited the most significant differences between PCOS and non-PCOS controls. DCA was associated with deposition index, fasting and postprandial insulin but was influenced by testosterone. CDCA and LCA combined with testosterone showed potential as biomarkers for the pathogenesis of PCOS.

**Conclusion:**

The circulating bile acid profile undergoes changes in PCOS. DCA is associated with deposition index, fasting and postprandial insulin and its level is influenced by testosterone. CDCA and LCA combined with testosterone have the potential to serve as biomarkers for the pathogenesis of PCOS.

## 1 Introduction

Polycystic ovary syndrome (PCOS) is one of the most common endocrine disorders among women of reproductive age, characterized by androgen excess, ovulatory dysfunction, and polycystic ovaries, often associated with metabolic abnormalities (1, 2). Insulin resistance and hyperandrogenism play pivotal roles in the pathogenesis of PCOS. An increasing body of evidence supports the crucial involvement of bile acids in metabolic diseases, with a growing number of studies exploring their relevance to PCOS. For instance, Li T. et al. demonstrated a potential association between improved levels of bile acids and steroid synthesis in PCOS mice following a probiotic response to intestinal flora (3). Yang Y.L. et al. found that CDCA-enhanced glucose metabolism in PCOS mice (4). Of particular interest, recent research has unveiled the role of bile acids in regulating reproductive function. Qi X. et al. confirmed that intestinal flora regulated IL-22 production via bile acids such as GDCA, which we also investigated in our study. Additionally, CDCA research has shown its significant effects on ovarian function and insulin sensitivity in PCOS patients (5). Furthermore, studies have reported that bile acids not only influenced the apoptosis of spermatocytes in male mice and sperm function (6) but also improved ovarian ischemia-reperfusion injury (7). The importance of bile acids in PCOS is becoming increasingly apparent, and identifying key differences in bile acids could hold potential as therapeutic targets for PCOS. Cholesterol is a substrate for the synthesis of steroid hormones. Bile acids (BAs) are amphiphilic steroid compounds produced by the liver through both the classic (neutral) and the alternative (acidic) pathway (8). Based on their production sites and raw materials, BAs can be categorized into two categories: primary bile acids and secondary bile acids. Recent studies have revealed that BAs not only impact the absorption of nutrients and vitamins in the body, but, more importantly, also act as an vital signaling molecules to regulate glucose and lipid metabolism (9, 10). In metabolic diseases such as obesity, diabetes and non-alcoholic steatohepatitis, it has been observed that total serum bile acid levels are significantly elevated, and each bile acid fraction undergoes significant changes (11–13). In obese patients, the level of cholic acid (CA) is particularly elevated, and both CA and CDCA show a positive correlation with insulin resistance (14). The BA signaling pathway plays a role in regulating various metabolic process, intestinal microbiota and even skeletal muscle growth. However, previous studies were mostly based on small samples, leading to varying research results regarding changes in bile acid fractions in patients with PCOS. The characteristics of the circulating bile acid profile and the relationship with glucose metabolism of PCOS have not been fully elucidated.

This study aimed to characterize and compare the absolute concentration and composition of circulating bile acids in patients with PCOS and non-PCOS controls, in order to identify the bile acids with specific differences. Additionally, we sought to assess their correlations with glucose metabolism characteristics and identify potential biomarkers for the pathogenesis of PCOS.

## 2 Materials and methods

### 2.1 Participants

The study enrolled 408 women with PCOS from the Endocrinology Department of Shanghai Renji Hospital, China, and 204 age- and BMI-matched normal cycling, non-hirsute and non-hyperandrogenemia women between 18–45 years of age. The recruitment period for the study spanned from January 2017 to December 2021.PCOS was diagnosed using the 2003 Rotterdam diagnostic criteria (15). Hyperandrogenism was determined based on serum testosterone levels. Impaired glucose regulation (IGR), was diagnosed according to the criteria set by the American Diabetes Association (16). Normal Glucose Tolerance (NGT) was defined as fasting glucose fasting glucose (FPG)<5.6 mmol/L and 2 h postprandial glucose (PPG)<7.8 mmol/L; IGR was defined as 5.6 mmol/L ≤ FPG<7.0 mmol/L and/or 7.8 mmol/L ≤ PPG<11.1 mmol/L. Pregnancy was excluded through a urine pregnancy test. All women underwent transvaginal or transanal ultrasonography for evaluating ovarian morphology and were screened through medical history assessment, including dietary habits, physical examination and laboratory evaluation. B-ultrasound showed normal gallbladder size, smooth gallbladder wall and clear bile. Subjects with a history of gastrointestinal surgery, tobacco or alcohol abuse, diabetes, coronary heart disease, abnormal renal function, active liver disease, chronic metabolic acidosis, chronic inflammatory disease or severe chronic gastrointestinal disease and premature ovarian failure were excluded. Additionally, women who had received glucocorticoids, antiandrogens, or oral contraceptives within the previous 30 days, or ovulation induction agents, anti-obesity medications, medication affecting bile acids metabolism, or insulin-sensitizing agents within the previous 60 days were also excluded.

All study evaluations and procedures were conducted in accordance with the guidelines of Helsinki Declaration on human experimentation. The study received approval from the ethics committee of Renji Hospital, Shanghai Jiao Tong University School of Medicine. (KY2021-191-B). Written informed consent was obtained from all patients prior to any study-related procedures.

### 2.2 Anthropometric measurements

The height and weight of each subject, while wearing light clothing, were measured using a digital scale and stadiometer, with measurements rounded to the nearest 0.1 cm and 0.1 kg, respectively. BMI was calculated as body weight (kg) divided by the square of the height (m).

### 2.3 Laboratory assays

All subjects fasted for 10 hours or more, and at 8:00 a.m. on the following day, 5 ml of fasting venous blood was collected. Subsequently, an oral glucose tolerance test (OGTT), was conducted, with venous blood collected 120 minutes after 85g of oral glucose to determine blood glucose and insulin levels. Additionally, fasting blood was collected to assess liver and kidney function as well as lipid levels. Luteinizing hormone (LH), follicle-stimulating hormone (FSH), testosterone (T), estradiol (E2), sex hormone binding globulin (SHBG), and anti-mullerian hormone (AMH) were measured from fasting venous blood collection on days 2-5 of menstruation (follicular phase). In subjects with amenorrhoea >3 months, measurements were taken randomly. Calculation of insulin resistance index: steady-state model insulin resistance index (HOMA-IR) = (Fins × FPG)/22.5 (Fins: fasting insulin; FBG: fasting blood glucose). The formula for free androgen index (FAI) was calculated: FAI= T (nmol/L)/SHBG (nmol/L) ×100. The Matsuda index was calculated by:


$$1000/\sqrt{\left(fasting\;glucose\left[mmol/l\right]\right)*\left(fasting\;insulin\left[mIU/L\right]\right)*\;\left(mean\;glucose\left[mmol/l\right]\right)*\left(mean\;insulin\left[mIU/L\right]\right)}$$

The insulinogenic index was calculated as (△INS_0-30_/△GLU_0-30_), and the deposition index (DI) is calculated by multiplying the Matsuda index and Insulinogenic index. Laboratory tests were performed by the Department of Biochemistry and Nuclear Medicine, Renji Hospital, Shanghai Jiao Tong University School of Medicine, and an ultrasound of uterus and both adnexa was performed by the Department of Ultrasound Medicine, Renji Hospital, Shanghai Jiao Tong University School of Medicine.

### 2.4 Serum bile acid profile assessment

Bile acids were quantified in serum samples that had been stored at −80°C using a LC-MS/MS platform, following a previously published method (17). Bile acids were separated using an ACQUITY BEH C18 column (1.7 μm, 100 mm × 2.1 mm internal dimensions) (Waters, Milford, MA). The column elution solvents consist of water + 0.01% formic acid (A) and acetonitrile/methanol (87:13) + 0.01% formic acid (B). The flow rate was 450 μL/min as follows: 0-1 min (5% B), 1-5 min (5-25% B), 5-15.5 min (25-40% B), 15.5-17.5 min (40-95% B), 17.5-19 min (95% B), 19-19.5 min (95-5% B), and 19.6-21 min (5% B). The raw data obtained from UPLC-MS were collected with multiple reaction monitor (MRM), and the cone and collision energy for each bile acid used the optimized settings from QuanOptimize application manager (Waters). Several internal standards were added to each experimental and process standard sample prior to injection into the mass spectrometer. The measure of platform variability was determined by calculating the median relative standard deviation (RSD) for the internal standards (18). Quantified bile acids contained six primary species, including CA, CDCA, glycocholic acid (GCA), taurocholic acid (TCA), glycochenodeoxycholic acid (GCDCA), taurochenodeoxycholic acid (TCDCA); and nine secondary species, including deoxycholic acid (DCA), ursodeoxycholic acid(UDCA), lithocholic acid(LCA), glycodeoxycholic acid (GDCA), taurodeoxycholic acid (TDCA), glycoursodeoxycholic acid (GUDCA), TUDCA, glycolithocholic acid (GLCA) and taurolithocholic acid (TLCA). The detection instruments are API3200MD triple quadrupole mass spectrometer (American ABSciex company) and Shimadzu 20AD liquid chromatograph (Japan Shimadzu company).

### 2.5 Statistical analysis

Statistical analysis was performed using SPSS version 25.0 (SPSS Inc., Chicago, IL, USA) and GraphPad Prism 8.0 (GraphPad Software, Inc., San Diego, CA). The distribution of each continuous variable was assessed using the Kolmogorov–Smirnov test. Data were then presented as mean ± SD or median (25th-75th interquartile range) for variables with non-normal distribution. To compare the differences between the two groups, The Student’s t-test and Mann-Whitney U test were employed. Correlation analysis was conducted using used Sperman’s correlation analysis. For multivariate statistical analysis to reflect differences between patients with PCOS and controls, SIMCA-P software v.14.1 (Umetrics AB, Umea, Sweden) was utilized. Principal component analysis (PCA) and orthogonal partial least squares -discriminant analysis (OPLS-DA) models were constructed based on the metabolomics data. The variable importance in the projection (VIP) of the first principal component obtained from the OPLS-DA analysis was determined. In the univariate analysis, metabolites with a VIP > 1.0 and P-value< 0.05 were considered significantly different. Moreover, the quality of the OPLS-DA model was assessed using standard parameters (R2X and Q2). Extreme gradient boosting trees (XGboost) were constructed using Python: xgboost 1.2.1 All hypotheses were tested using a two-sided hypothesis. A P-value of<0.05 was considered statistically significant.

## 3 Results

### 3.1 Characteristics of the study subjects

**Table 1** summarizes the clinical baseline characteristics of study participants. Body mass index-matched women with PCOS and controls were enrolled to mitigate the influence of obesity on the bile acid profiles in individuals with PCOS. Age, BMI, liver function, triglycerides, total cholesterol, and low-density lipoprotein cholesterol showed no significant differences between the two groups(p>0.05). We also measured Anti-Müllerian hormone (AMH) and average ovary volume, as the circulating concentration of AMH in women reflects the number of follicles remaining in the ovary (19) and ovary volume can indirectly reflect an increased number of immature follicles in the ovary of PCOS. Individuals with PCOS exhibited significantly higher serum luteinizing hormone (LH) level, luteinizing hormone/follicle-stimulating hormone (FSH) ratio, testosterone level, androstenedione level, free androgen index value, AMH, average ovary volume(p<0.05). Meanwhile, individuals with PCOS had lower level of serum FSH, sex hormone binding globulin (SHBG) and high-density lipoprotein cholesterol(p<0.05), compared with healthy controls. Two-hour postprandial glucose and insulin were significantly higher in women with PCOS(p<0.001). Matsuda index was significantly lower in PCOS(p<0.05).

**Table 1:** Clinical baseline characteristics of study participants.

Variables	CON	PCOS	P value^a,b^
*N*	*204*	*408*	
Age, years	27.000(24.000,31.000)	27.000(24.425,31.000)	0.888
BMI, kg/m^2^	24.683(20.957,29.297)	24.609(21.368,28.006)	0.659
liver function			
ALT, U/L	24.239 ± 22.900	24.075 ± 23.717	0.935
AST, U/L	23.083 ± 19.054	22.262 ± 16.528	0.596
lipid metabolism			
TG, mmol/L,	0.990(0.760,1.430)	1.060(0.870,1.360)	0.174
TC, mmol/L	4.550(3.930,5.150)	4.560(4.030,5.140)	0.509
HDL-c, mmol/L	1.430(1.120,1.770)	1.290(1.140,1.540)	0.022^a^
LDL-c, mmol/L	2.460(2.020,3.010)	2.630(2.150,3.140)	0.061
sex hormone			
LH/FSH	1.051 ± 0.754	1.384 ± 0.963	<0.001^b^
LH, IU/L	6.964 ± 5.134	8.533 ± 6.175	0.001 ^b^
FSH, IU/L	7.448 ± 4.497	6.505 ± 2.715	0.007 ^b^
E2, pmol/L	225.371 ± 192.913	184.246 ± 209.727	0.020 ^a^
T, nmol/L	1.540(1.060,1.960)	2.210(1.600,2.740)	<0.001 ^b^
SHBG, nmol/L	38.300(29.600,53.100)	29.000(19.100,44.100)	<0.001 ^b^
A2, ug/dl	3.063 ± 1.877	4.196 ± 2.365	<0.001 ^b^
AMH	3.586 ± 2.431	8.865 ± 5.741	<0.001 ^b^
FAI	3.961(2.643,5.290)	7.855(4.016,12.021)	<0.001 ^b^
glucose metabolism			
0’PG, mmol/L	4.855 ± 0.666	4.953 ± 0.702	0.110
120’PG, mmol/L	6.552 ± 1.873	7.128 ± 1.838	<0.001 ^b^
0’Ins, mIU/L	11.817 ± 9.622	11.165 ± 9.049	0.428
120’Ins, mIU/L	73.979 ± 59.478	93.181 ± 73.149	<0.001 ^b^
HOMA-IR	2.642 ± 2.420	2.513 ± 2.237	0.528
Deposition Index	17.787 ± 23.749	18.072 ± 38.527	0.927
Matsuda index	10.561 ± 7.137	9.350 ± 6.037	0.047 ^a^
ovary volume			
Average ovary volume, mm^3^	6.621 ± 3.028	11.005 ± 6.167	<0.001 ^b^

Normally distributed data are expressed as mean ± SD, and independent samples t-test was used for comparison between groups. Non-normally distributed data are expressed as median (25th and 75th quartiles), and the Mann-Whitney test was used for comparison between groups. BMI, body mass index; ALT, alanine aminotransferase; AST, aspartate aminotransferase; TG, triglycerides; TC, total cholesterolc; HDL-c, cholesterol associated with high density lipoproteins; LDL-c, cholesterol associated with low density lipoproteins; LH, luteinizing hormone; FSH, follicle stimulating hormone; E2, Estradiol; T, testosterone; SHBG, sex hormone-binding globulin; A2, androstenedione; AMH, anti-mullerian hormone; FAI, free androgen index 0’PG; fasting plasma glucose; 0’ins, fasting insulin; HOMA-IR, homeostasis model assessment of insulin resistance. ^a^: P<0.05; ^b^: P<0.01.

### 3.2 The analysis of bile acid profile in serum in patients with PCOS

**Figure 1:**
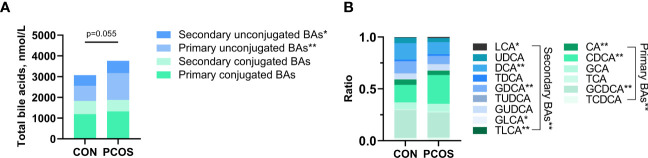
Comparison of bile acid pools **(A)** and relative fractions of bile acids **(B)** in the PCOS and Control groups. Relative fractions of bile acids were the ratio obtained by dividing the bile acid concentration by the total bile acid. Independent samples t-test was used for comparison between groups. (*p<0.05, ** p<0.01).

The serum bile acid profiles of PCOS patients and age- and BMI-matched controls were obtained. Fifteen bile acid fractions, comprising primary unconjugated bile acids, secondary unconjugated bile acids, primary conjugated bile acids, and secondary conjugated bile acids were identified. While there was a trend towards higher total bile acid levels in PCOS compared to controls, this difference did not reach statistical significance (p=0.055). However, both primary unconjugated bile acids and secondary unconjugated bile acids were found to be significantly higher in PCOS patients (both p<0.05) (**Figure 1A**). Regarding the analysis of the ratio of 15 bile acid metabolites to total bile acids, PCOS patients exhibited relatively higher percentages of LCA and CDCA, while their percentages of CA, DCA, GDCA, GLCA, TLCA and GCDCA were relatively lower, all showing statistical significance (all p<0.05). In contrast, the percentages of the other seven bile acid metabolites did not differ significantly between the two groups (**Figure 1B** and **Table S1**).

### 3.3 Identification of significantly different metabolites associated with PCOS

**Figure 2:**
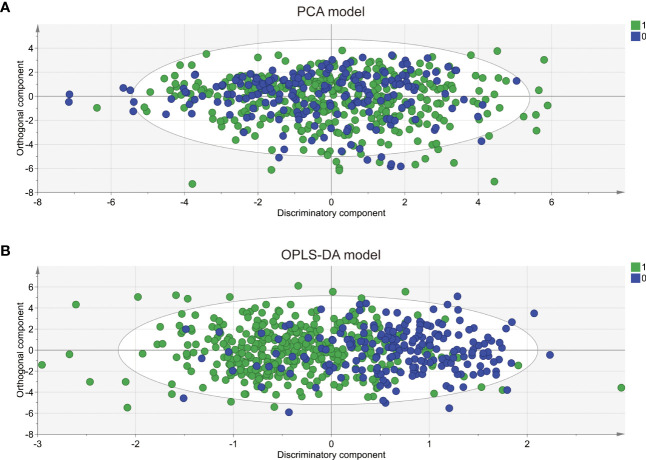
PCA and OPLS-DA score plots derived from bile acid metabolomic profiles comparing PCOS patients and controls. Principal component analysis (PCA) **(A)** and orthogonal partial least squares discriminant analysis (OPLS-DA) **(B)** were performed for the controls (circle in green) and the PCOS patients (circle in blue). The PCA model was unable to distinguish two completely separate groups (R2X(cum) 0.844, Q2 0.41; while the OPLS-DA model clearly discriminated between the two groups but incompletely(R2X(cum) 0.775, Q2(cum) 0.274).0: CON; 1: PCOS.

To comprehensively compare the bile acid metabolomic profile between PCOS patients and non-PCOS controls, we employed the PCA and OPLS-DA model to assess the degree of diversity. The PCA model demonstrated an inability to distinctly separate the two groups (R2X(cum) 0.844, Q2(cum) 0.41; **Figure 2A**), while the OPLS-DA model exhibited clearer discrimination, though not entirely conclusive (R2X(cum) 0.775, Q2(cum) 0.274, **Figure 2B**). Considering the inherent variability in human biological samples (20), the R2X value being above 0.5 (with a relatively low Q2 value of at 0.274) was deemed acceptable. To further validate the prediction model, we conducted permutation tests. The Q2 intercept value falling below 0 indicated a reliable and non-overfitted model. The OPLS-DA analysis identified five bile acid metabolites (CDCA, LCA, GDCA, DCA and TCDCA) with VIP values greater than 1 and exhibiting significant differences (P<0.05) in the univariate analysis between PCOS and controls (**Table 2**). We subsequently confirmed that the level of these five metabolites were significantly higher in PCOS patients compared to controls using t-test (P<0.01). Notably, CDCA displayed the most pronounced elevation in PCOS and was determined to hold valuable significance in the bile acid composition of PCOS when compared with non-PCOS controls (VIP score=2.04644, p<0.001).

**Table 2:** Bile acid metabolites in serum samples differentiating PCOS women from controls (VIP scores).

Parameter	VIP score	CON	PCOS	P value	PCOS vs CON
CDCA	2.04644	551.885 ± 726.902	1123.086 ± 2631.657	<0.001	↑
LCA	1.22478	12.453 ± 10.836	35.836 ± 225.476	<0.001	↑
GDCA	1.15657	403.968 ± 600.944	276.679 ± 383.015	0.002	↓
DCA	1.02392	383.505 ± 334.353	413.981 ± 1718.786	0.002	↑
TCDCA	1.01146	76.113 ± 71.501	108.929 ± 161.206	0.006	↑

Normally distributed data are expressed as mean ± SD, and independent samples t-test was used for comparison between groups. Arrows show whether the bile acid metabolites in PCOS women were present at higher or lower concentrations than in CON women. CDCA, chenodeoxycholic acid; LCA, lithocholic acid; GDCA, glycodeoxycholic acid; DCA, deoxycholic acid; TCDCA, taurochenodeoxycholic acid.

### 3.4 Associations of five differential bile acid metabolites with glucose metabolism in PCOS patients

In addition to their well-established role in promoting lipid absorption, Bile Acids (BAs) are also implicated in glucose metabolism and the secretion of glucoregulatory hormones (21, 22). Previous studies have shown that alterations in CDCA levels are correlated with reduced insulin sensitivity (14). Hyperandrogenism and insulin resistance are the primary features of PCOS, and they are risk factors for the development of abnormal glucose metabolism. To determine whether specific bile acid metabolites are related to glucose metabolism in PCOS, we analyzed the correlations between candidate bile acid metabolites and glucose metabolism characteristics, while adjusting for testosterone levels (**Table 3**). Before adjustment, we found significant positive correlations between CDCA and multiple glucose and lipid metabolism parameters, as well as hepatic inflammation. Additionally, DCA was correlated with deposition index, fasting and postprandial insulin levels. However, no significant correlations were observed between other candidate metabolites and metabolism in patients with PCOS (**Table S2**). After adjusting for testosterone levels, CDCA and GDCA remained correlated with postprandial glucose levels, while LCA showed correlation with postprandial insulin (**Table 3**). Our results suggested that the effect of bile acids on glucose metabolism in PCOS might be weaker than that of testosterone. Furthermore, we conducted a subgroup analysis of glucose metabolism to explore the relationship between bile acid and glucose metabolism. The baseline characteristics of the PCOS patients with NGT and IGR were presented in **Table S3**. PCOS patients with IGR displayed higher serum testosterone levels. As shown in **Table S4**, PCOS patients with IGR had lower levels of DCA, and no significant changes in other bile acids were observed. DCA also showed a correlation with deposition index, fasting and postprandial insulin in PCOS. These findings suggest that the influence of bile acids on glucose metabolism in PCOS might be weaker compared to that of androgens.

**Table 3:** Correlations between candidate bile acid metabolites and glucose metabolism adjusted testosterone in PCOS patients.

	CDCA	LCA	GDCA	DCA	TCDCA
0’ PG	0.062	-0.05	-0.004	0.017	0.01
120’ PG	0.094 ^a^	-0.036	-0.09 ^a^	0.021	-0.01
0’ Ins	0.02	0.063	-0.023	-0.023	0.009
120’ Ins	0.061	0.102 ^a^	-0.075	0.012	0.037
HOMA-IR	0.027	0.046	-0.027	-0.024	0.006
Deposition Index	-0.034	-0.006	-0.026	-0.014	0.005
Matsuda index	-0.048	-0.042	-0.004	0.011	-0.062

a : P<0.05. Adjusted testosterone.

### 3.5 The difference of the levels of bile acids in the PCOS with non-HA or HA and correlation between differential bile acid and glucose metabolism in two groups

In the investigation of the association between bile acids and glucose metabolism, we observed a considerable attenuation of the correlation after adjusting for testosterone, which piqued our interest. Consequently, we proceeded to subgroup the PCOS patients based on the presence or absence of hyperandrogenism (non-HA or HA). The clinical baseline characteristics of these two groups were presented in **Table S5**. Notably, PCOS patients with HA exhibited more pronounced abnormalities in glucose metabolism compared to those without HA. **Table 4** illustrates the disparities in bile acid levels between the two groups, revealing significantly higher concentrations of CA and CDCA in PCOS patients with HA. It’s of interest that the level of CDCA was also elevated in the comparison between PCOS and non-PCOS.

**Table 4:** Comparison of the concentration of 15 bile acid in the PCOS with non-HA or HA groups.

Variables	non-HA	HA	P value ^a,b^
*N*	*70*	*140*	
CA	107.21(84.37-154.09)	126.99(94.32-209.72)	0.031^a^
DCA	253.13(116.85-378.02)	207.31(57.16-434.29)	0.395
CDCA	601.57(381.20-1049.40)	812.40(445.62-1003.90)	0.026^a^
UDCA	86.15(33.54-135.15)	95.89(35.64-201.32)	0.170
LCA	20.81(12.00-30.48)	18.1(10.59-30.00)	0.214
GCA	96.22(63.78-166.13)	130.08(64.68-242.50)	0.146
GLCA	5.47(2.73-10.00)	4.72(2.03-10.00)	0.611
GDCA	136.70(88.78-234.53)	126.23(32.41-255.51)	0.403
GCDCA	635.45(343.86-1020.38)	628.07(335.66-1149.85)	0.916
GUDCA	115.84(55.73-239.78)	106.66(50.15-251.93)	0.881
TCA	11.41(7.06-37.05)	18.57(8.50-32.99)	0.372
TLCA	0.98(0.43-2.00)	1(0.29-2.00)	0.727
TDCA	18.89(10.00-34.20)	17.05(8.10-34.93)	0.426
TCDCA	56.37(30.84-145.38)	61.07(29.89-110.35)	0.731
TUDCA	3.78(2.10-8.14)	4.01(2.03-7.42)	0.969

Normally distributed data are expressed as mean ± SD, and independent samples t-test was used for comparison between groups. Non-normally distributed data are expressed as median (25th and 75th quartiles), and the Mann-Whitney test was used for comparison between groups.

^a^: P<0.05; ^b^: P<0.01.

The correlations between the two groups displayed distinct patterns, as evident from **Table 5**. In PCOS patients with HA, there was a significant association between CA and the deposition index, a relationship not observed in PCOS patients without HA. Likewise, DCA was significantly associated with deposition index, fasting and postprandial insulin, which was not found in PCOS patients without HA.

**Table 5:** Correlations between differential bile acid metabolites and glucose metabolism in PCOS patients with non-HA or HA.

	non-HA			HA		
	CDCA	CA	DCA	CDCA	CA	DCA
0’ PG	-0.029	-0.001	0.004	0.053	0.08	-0.054
120’ PG	0.101	0.120	-0.090	0.065	0.125	-0.011
0’ Ins	-0.064	-0.054	-0.060	0.019	0.108	-0.106^a^
120’ Ins	-0.050	-0.028	-0.117	0.014	0.071	-0.099^a^
HOMA-IR	-0.051	-0.028	-0.045	0.022	0.114	-0.049
Deposition Index	-0.089	-0.053	0.127	-0.079	-0.154^a^	-0.092^a^
Matsuda index	0.044	-0.014	0.086	-0.031	-0.122	0.013

^a^: P<0.05.

### 3.6 The candidate value of bile acid in serum for PCOS

**Figure 3:**
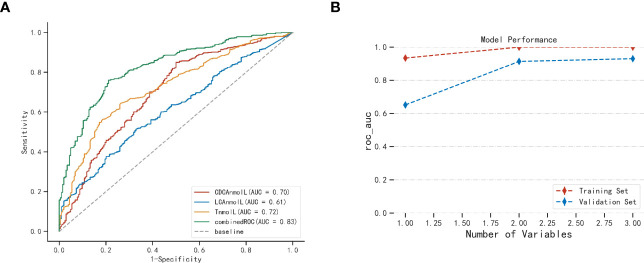
The candidate value of bile acid in serum for PCOS **(A)** and XGboost validation of model performance **(B)**. AUC, Area under the curve. Combined ROC: CDCA、LCA combined testosterone.

Our aim is to investigate the predictive value of bile acid metabolites for PCOS. **Figure 3A** and **Table S6** illustrate the areas under the ROC curve for CDCA and LCA, which were 0.669 and 0.612, respectively. Additionally, the areas under the ROC curve of CA and DCA were 0.578 and 0.544, respectively, indicating that CA and DCA were not effective predictors of PCOS. On the other hand, the area under the ROC curve for testosterone was 0.725. To determine if bile acid metabolites could enhance the predictive accuracy of PCOS compared to the conventional index of testosterone alone, we combined CDCA, LCA, and testosterone for prediction. Remarkably, the combined ROC curve demonstrated an increased area under the curve, reaching 0.827. The efficacy of this combined ROC was validated through the Delong test, showing a significantly higher predictive value than individual indicators (p<0.01, **Table S7**).

XGboost was employed with five cross-validations to internally verify the model fit (23). **Figure 3B** illustrates the use of XGboost for variable set scoring. During the scoring process, the variables in the set [‘T’, ‘CDCA’, ‘LCA’] were added sequentially (following the importance ranking obtained from the *a priori* estimation), resulting in the best performance when all three variables were included. Thus, the optimal set of variables for predictive modeling of PCOS was [‘T’, ‘CDCA’, ‘LCA’]. This cross-validation highlights the significance of selected CDCA, LCA, and T parameters in the predictive modeling of PCOS.

## 4 Discussion

In this study, we employed a large sample of clinical data to investigate the comprehensive alterations in the circulating bile acid profile in PCOS. Our analysis revealed five differential bile acids that exhibited significant changes in PCOS. In the correlation analyses we found that DCA was associated with deposition index, fasting and postprandial insulin but was influenced by adjusting for testosterone. Moreover, we identified CDCA and LCA as candidate bile acid metabolites that combined with testosterone demonstrated diagnostic value for PCOS.

PCOS is a clinically heterogeneous disorder, and its routine diagnosis relies on the 2003 Rotterdam Criteria. Although it can be easily diagnosed through featured ultrasound changes in the ovary and menstrual irregularity, the current diagnostic criteria do not encompass the underlying differences in biology associated with different forms of PCOS (24). The need for a more concise diagnosis of PCOS is growing since in clinical practice, there are often situations that are confused with PCOS. For example, 20% of the normal population may present with ovarian polycystic manifestations (25). Congenital adrenal hyperplasia or hypothyroidism can also present with hyperandrogenism and menstrual irregularity. Our study attempted to explore bile acid biomarkers of pathogenesis of PCOS for precise diagnosis, considering the emerging interest in intestinal flora and bile acids in etiologic studies of PCOS (26).

By using OPLS-DA model and VIP score (27), we identified five bile acid metabolites (in order of CDCA, LCA, GDCA, DCA, and TCDCA) that exhibited significantly differences in PCOS compared to the non-PCOS controls. Our findings indicated that CDCA played the most prominent role in distinguishing PCOS from the control group. In the bile acid synthesis pathway, CDCA is predominantly produced through the alternative pathway (8). Elevated CDCA levels may signify the activation of the alternative bile acid synthesis pathway in PCOS patients. This activation can be a pathological state leading to impaired synthesis of the classical pathway, a phenomenon also observed in NAFLD (28). This may explain the cause of elevated CDCA in PCOS. However, further experimental evidence is needed to substantiate this hypothesis. LCA is produced by CDCA in the presence of intestinal flora (29), and we found that LCA also contributed to distinguishing PCOS from control. Bile acid profiles in patients with hypothyroidism have been reported (30). Unlike patients with PCOS, patients with hypothyroidism showed decreased CDCA and LCA and increased levels of DCA. In contrast, PCOS patients exhibited elevated levels of CDCA and LCA. These findings suggest that the bile acid synthesis pathway may undergo distinct changes in the two diseases. Bile acids may be able to differentiate between PCOS and hypothyroidism, which contributed to precise diagnosis of PCOS.

In correlation analysis, prior to correcting for confounders, we observed a significant association between elevated CDCA and multiple characteristics of glucolipid metabolism. Testosterone was adjusted for, as it is believed to play an important role in insulin resistance and abnormal glucose metabolism in PCOS (31). Following the correction, altered bile acids CDCA and LCA remained correlated with postprandial glucose and insulin, while the correlations with other characteristics diminished. Further subgroup analyses of glucose metabolism were conducted, revealing that only DCA exhibited differences in bile acid levels between the two groups. Previous studies reported that DCA was associated with insulin secretory function (32).The role of bile acids in glucose metabolism abnormalities has been clearly defined in non-PCOS. However, in PCOS, our results suggest that androgens may play a more important role in glucose metabolism abnormalities compared to bile acids. Nonetheless, it is also plausible that an interaction exists between androgens and bile acid. To explore this, we performed a subgroup analysis focusing on androgens. In PCOS patients with HA, we found elevated levels of bile acid CA and CDCA. CDCA was most prominently elevated in both PCOS and non-PCOS groups, implying that CDCA could be an important role in the pathogenesis of hyperandrogenism in PCOS. Future research can focus on this point. Separate correlation analyses for the HA and non-HA groups indicated that CA was correlated to deposition index in PCOS with HA and DCA was correlated to deposition index, fasting and postprandial insulin. In the analyses of correlation in all PCOS patients, before adjusting for androgens, DCA also showed a correlation with the deposition index. DCA is produced from CA by gut microbiota. The correlation between DCA and insulin secretion and function may be related to the role of gut flora. DCA was associated with deposition index, fasting and postprandial insulin in PCOS patients with HA which was different in non-HA group. Our results also suggest that after adjusting for androgen and removing the role of androgens on gut flora, DCA, a metabolite of the intestinal flora, was no longer associated with glucose metabolism in all PCOS patients. Qi et al. reported a significantly positive correlation between serum DCA levels and B. vulgatus levels, with B. vulgatus being markedly elevated in the gut microbiota of individuals with PCOS (5). Previous studies have also suggested a potential role of androgens in the alteration of gut flora in PCOS (33). Thus, our results propose that androgens may influence changes in DCA through elevated B. vulgatus, which in turn affects insulin secretion, but this requires further verification through additional experiments.

We also observed a significant elevation in the total bile acid level in PCOS compared to the normal population, probably because the total cholesterol level did not differ between the two groups. Interestingly, unlike a previous small sample study, we delved into the proportion of bile acid fractions within the total bile acids. Analyzing the proportion of bile acid fractions aids in understanding changes in the bile acid synthesis pathway, and the upregulation of alternative bile acid synthesis pathways has been suggested as a therapeutic target in NAFLD-related studies (28). Our investigation revealed that within the composition of the bile acid pool, primary unconjugated bile acids and secondary unconjugated bile acids were relatively elevated in PCOS. In a previous study, elevated primary conjugated bile acids were associated with hyperandrogenism in PCOS (34). The disparity in study findings may be attributed to sample size or population bias. Our study involved a more accurate determination of the 15 fractions of bile acids, revealing that in the analysis of the ratio of 15 bile acid metabolites to total bile acids, the percentages of LCA and CDCA to total bile acids were relatively higher, while the percentages of CA, DCA, GDCA, GLCA, TLCA and GCDCA to total bile acids were relatively lower in PCOS patients. These findings might account for the significant changes observed in the unconjugated bile acid fraction in PCOS. The decrease in the CA ratio and the increase in the CDCA ratio might suggest alterations in the bile acid synthesis pathway in PCOS, although further experiments are required to elucidate this hypothesis.

Based on the elucidation of the bile acid profile characteristics of PCOS, we explored potential bile acid biomarkers of the pathogenesis of PCOS. Comparing bile acid ratios between PCOS and normal populations, we observed significant changes in bile acid fractions, particularly CDCA and LCA, in PCOS. Furthermore, the OPLS-DA model’s multifactorial analysis revealed the significant contribution of CDCA and LCA in distinguishing PCOS from non-PCOS controls. Additionally, we found that the combination of CDCA, LCA and testosterone served as a more effective predictor for PCOS compared to using testosterone alone. CDCA and LCA are the primary ligands of bile acid receptors, and they can influence hepatic fat deposition, fat oxidation, insulin secretion, energy balance and other metabolism through bile acid FXR or TGR5 signaling (35–38). These findings contribute to our understanding of signaling pathways involving bile acid, and future research should focus on investigating downstream molecules in these pathways.

There are certain limitations to our study. Firstly, it is important to note that our study is a cross-sectional study, and therefore, the exact role of bile acids in the etiology of PCOS requires further investigation. Additionally, the relationship between bile acids and gut flora needs to be more extensively explored. Furthermore, the potential treatment strategy based on bile acids should to be clarified through randomized controlled trials in the future.

Nevertheless, our study has successfully established a comprehensive profile of bile acid in PCOS. We have also hypothesized that the relationship between DCA and insulin secretory function may be influenced by androgenic effects on gut flora. We have identified potential metabolic biomarkers that can be utilized to distinguish PCOS women and non-PCOS controls, thereby potentially enhancing the diagnosis and management of PCOS.

## Data availability statement

The raw data supporting the conclusions of this article will be made available by the authors, without undue reservation.

## Ethics statement

The studies involving humans were approved by the ethics committee of Renji Hospital, Shanghai Jiao Tong University School of Medicine. The studies were conducted in accordance with the local legislation and institutional requirements. The participants provided their written informed consent to participate in this study.

## Author contributions

JY and TT designed research; JY and YZ conducted data analysis. YCZ, YZ, YL, and SL carried data collection; TT and WL supervised research; JY and TT wrote the paper. All authors contributed to the article and approved the submitted version.
