# Supplementary material for: Circulating bile acid profile characteristics in PCOS patients and the role of bile acids in predicting the pathogenesis of PCOS
Source: Front Endocrinol (Lausanne). 2023 Aug 24;14:1239276. doi: 10.3389/fendo.2023.1239276 (PMC10484098; doi:10.3389/fendo.2023.1239276)
Supplement: Supplementary file 1 [file DataSheet_1.docx]

Supplementary Material

# Supplementary Tables

**Table S1.** **Comparison of the ratio of 15 bile acid fractions to total BAs in the PCOS and Control groups.**

| Variables | CON | PCOS | P value ^a, b^ |
| --- | --- | --- | --- |
| N | 204 | 408 |  |
| CA | 0.033(0.018,0.059) | 0.025(0.014,0.049) | <0.001^b^ |
| DCA | 0.141(0.063,0.241) | 0.096(0.028,0.174) | <0.001^b^ |
| CDCA | 0.134(0.072,0.234) | 0.250(0.167,0.363) | <0.001^b^ |
| UDCA | 0.032(0.016,0.058) | 0.029(0.015,0.051) | 0.131 |
| LCA | 0.004(0.001,0.008) | 0.005(0.001,0.012) | 0.015^a^ |
| GCA | 0.052(0.034,0.079) | 0.058(0.037,0.095) | 0.119 |
| GLCA | 0.002(0.001,0.005) | 0.002(0.000,0.004) | 0.018^a^ |
| GDCA | 0.100(0.057,0.161) | 0.064(0.018,0.116) | <0.001^b^ |
| GCDCA | 0.271(0.194,0.346) | 0.226(0.161,0.313) | 0.001^b^ |
| GUDCA | 0.038(0.022,0.061) | 0.040(0.023,0.072) | 0.388 |
| TCA | 0.006(0.003,0.011) | 0.007(0.003,0.013) | 0.124 |
| TLCA | 0.000(0.000,0.001) | 0.000(0.000,0.001) | 0.004^b^ |
| TDCA | 0.014(0.006,0.025) | 0.011(0.004,0.025) | 0.312 |
| TCDCA | 0.023(0.013,0.036) | 0.024(0.014,0.039) | 0.083 |
| TUDCA | 0.001(0.001,0.002) | 0.001(0.001,0.003) | 0.314 |

**Relative fractions of bile acids were the ratio obtained by dividing the bile acid concentration by the total bile acid. Independent samples t-test was used for comparison between groups.** **^a^: P<0.05, ^b^: P<0.01.**

**Table S2. Correlations between candidate bile acid metabolites and hepatic inflammation, Blood lipids and glucose metabolism in PCOS patients without adjustment.**

|  | CDCA | LCA | GDCA | DCA | TCDCA |
| --- | --- | --- | --- | --- | --- |
| ALT | 0.190^b^ | -0.006 | -0.048 | -0.033 | -0.046 |
| AST | 0.112^a^ | 0.001 | 0.020 | -0.006 | 0.034 |
| TG | 0.115^a^ | 0.022 | 0.053 | -0.006 | 0.098 |
| TC | 0.069 | 0.103 | -0.017 | -0.035 | 0.003 |
| HDL-c | -0.085 | -0.049 | -0.035 | 0.067 | -0.066 |
| LDL-c | 0.113^a^ | 0.056 | -0.011 | -0.051 | -0.004 |
| 0’PG | 0.135^a^ | -0.095 | -0.017 | -0.052 | -0.062 |
| 120’PG | 0.212^b^ | 0.015 | -0.084 | -0.081 | -0.027 |
| 0’ ins | 0.172^b^ | 0.000 | -0.026 | -0.099^a^ | -0.030 |
| 120’ Ins | 0.206^b^ | 0.059 | -0.097 | -0.107^a^ | -0.025 |
| HOMA-IR | 0.182^b^ | -0.02 | -0.016 | -0.091^a^ | -0.040 |
| Deposition Index | -0.162^b^ | 0.045 | 0.061 | 0.105^a^ | 0.078 |
| Matsuda index | -0.225^b^ | -0.010 | 0.043 | 0.056 | 0.024 |

^a^: P < 0.05，^b^: P < 0.01.

**Table S3. Clinical baseline characteristics of PCOS with NGT or IGR.**

| Variables | NGT | IGR | P value ^a, b^ |
| --- | --- | --- | --- |
| *N* | *180* | *90* |  |
| Age, years | 27.47+4.48 | 27.16+4.26 | 0.562 |
| BMI, kg/m^2^ | 26.88+4.75 | 26.99+5.11 | 0.201 |
| ***liver function*** |  |  |  |
| ALT, U/L | 14.50(10.00-24.75) | 28.50(15.00-36.25) | <0.001^b^ |
| AST, U/L | 17.00(14.00-20.00) | 22.50(16.00-33.25) | <0.001 ^b^ |
| ***lipid metabolism*** |  |  |  |
| TG, mmol/L, | 1.02(0.78-1.73) | 1.72(1.24-2.14) | 0.001^b^ |
| TC, mmol/L | 3.04+0.9 | 4.97+1.13 | 0.015^a^ |
| HDL-c, mmol/L | 1.95+0.38 | 1.05+0.31 | <0.001^b^ |
| LDL-c, mmol/L | 2.01+0.80 | 2.92+0.85 | 0.007^b^ |
| ***sex hormone*** |  |  |  |
| LH, IU/L | 5.47(2.15-10.1) | 5.66(2.98-10.11) | 0.073 |
| FSH, IU/L | 6.79+2.80 | 6.53+2.70 | 0.535 |
| E2, pmol/L | 193.00(128.00-256.00) | 169.00(100.00-280.20) | 0.189 |
| T, nmol/L | 1.29+1.00 | 2.73+0.94 | 0.002^b^ |
| SHBG, nmol/L | 42.80(22.13-43.96) | 22.40(14.60-32.20) | <0.001^b^ |
| AMH | 5.60(3.02-10.20) | 4.93(2.38-8.56) | 0.502 |
| FAI | 7.90(5.52-12.13) | 11.46(7.70-20.63) | <0.001^b^ |
| ***glucose metabolism*** |  |  |  |
| 0’PG, mmol/L | 4.15+0.39 | 6.05+1.08 | <0.001^b^ |
| 120’PG, mmol/L | 5.82+1.03 | 10.00+2.15 | <0.001^b^ |
| 0’Ins, mIU/L | 8.66+5.97 | 15.90+10.15 | <0.001^b^ |
| 120’Ins, mIU/L | 65.60+50.14 | 143.88+90.90 | <0.001^b^ |
| HOMA-IR | 1.55(0.90-2.42) | 3.68(2.18-5.67) | <0.001^b^ |
| Deposition Index | 14.34(8.60-24.08) | 4.07(2.20-9.91) | <0.001^b^ |
| Matsuda index | 9.91(6.37-15.51) | 4.1(2.85-7.73) | <0.001^b^ |

Normally distributed data are expressed as mean ± SD, and independent samples t-test was used for comparison between groups. Non-normally distributed data are expressed as median (25th and 75th quartiles), and the Mann-Whitney test was used for comparison between groups.

^a^ : P<0.05; ^b^: P<0.01.

**Table S4. Comparison of the concentration of 15 bile acid in the PCOS with NGT or IGR groups.**

| Variables | NGT | IGR | P value ^a, b^ |
| --- | --- | --- | --- |
| *N* | *180* | *90* |  |
| CA | 119.09(87.8-188.05) | 133.63(100.40-198.37) | 0.093 |
| DCA | 362.74+297.90 | 277.40+295.26 | 0.039^a^ |
| CDCA | 692.80(410.95-1220.54) | 854.31(438.85-1461.19) | 0.424 |
| UDCA | 95.85(35.22-195.78) | 95.85(35.22-195.78) | 0.902 |
| LCA | 18.73(10.90-30.00) | 18.22(11.44-30.00) | 0.909 |
| GCA | 114.76(63.53-218.72) | 141.72(70.41-265.01) | 0.151 |
| GLCA | 4.76(1.84-10.00) | 5.05(2.38-10.00) | 0.909 |
| GDCA | 136.70(62.00-271.23) | 107.74(24.67-225.94) | 0.094 |
| GCDCA | 107.74(24.67-225.94) | 670.85(312.55-1315.20) | 0.747 |
| GUDCA | 112.87(51.39-296.47) | 96.31(49.26-215.78) | 0.340 |
| TCA | 14.67(7.67-29.48) | 23.90(8.93-39.23) | 0.061 |
| TLCA | 1.31+1.33 | 1.32+1.16 | 0.913 |
| TDCA | 18.89(9.71-34.93) | 15.09(7.38-34.20) | 0.474 |
| TCDCA | 57.79(31.01-110.16) | 68.46(28.00-120.96) | 0.586 |
| TUDCA | 3.91(1.93-7.58) | 4.77(2.36-8.74) | 0.797 |

Normally distributed data are expressed as mean ± SD, and independent samples t-test was used for comparison between groups. Non-normally distributed data are expressed as median (25th and 75th quartiles), and the Mann-Whitney test was used for comparison between groups.

^a^ : P<0.05; ^b^: P<0.01.

**Table S5. Clinical baseline characteristics of PCOS with non-HA or HA.**

| Variables | non-HA | HA | P value ^a, b^ |
| --- | --- | --- | --- |
| *N* | *70* | *140* |  |
| Age, years | 26.39+4.34 | 26.79+5.34 | 0.610 |
| BMI, kg/m^2^ | 24.89+4.70 | 24.62+4.80 | 0.562 |
| ***liver function*** |  |  |  |
| ALT, U/L | 16.79+10.98 | 22.49+12.19 | 0.002^b^ |
| AST, U/L | 20.22+7.17 | 21.17+8.86 | 0.067 |
| ***lipid metabolism*** |  |  |  |
| TG, mmol/L, | 0.98(0.65-1.94) | 1.40(1.01-1.97) | 0.012^a^ |
| TC, mmol/L | 4.65+0.96 | 4.92+0.99 | 0.060 |
| HDL-c, mmol/L | 1.97+0.34 | 1.09+0.34 | 0.001^b^ |
| LDL-c, mmol/L | 2.02+0.82 | 2.91+0.88 | 0.03^a^ |
| ***sex hormone*** |  |  |  |
| LH, IU/L | 5.27(3.79-12.25) | 7.42(3.68-12.55) | 0.381 |
| FSH, IU/L | 6.18(4.47-7.74) | 6.45(5.22-7.79) | 0.545 |
| E2, pmol/L | 225.68+155.62 | 214.90+151.80 | 0.601 |
| T, nmol/L | 2.11+0.86 | 2.97+0.85 | <0.001^b^ |
| SHBG, nmol/L | 49.70(33.00-76.80) | 23.50(15.65-35.00) | <0.001^b^ |
| AMH | 5.13(3.05-9.12) | 5.30(2.78-10.08) | 0.936 |
| FAI | 4.23(2.80-5.88) | 10.82(7.98-17.25) | <0.001^b^ |
| ***glucose metabolism*** |  |  |  |
| 0’PG, mmol/L | 4.78+0.98 | 4.82+0.60 | 0.377 |
| 120’PG, mmol/L | 6.00+1.54 | 7.37+2.66 | 0.001^b^ |
| 0’Ins, mIU/L | 7.38+5.25 | 13.20+9.26 | <0.001^b^ |
| 120’Ins, mIU/L | 66.23+58.34 | 104.06+82.03 | <0.002^b^ |
| HOMA-IR | 1.09(0.75-2.25) | 2.26(1.31-4.11) | <0.003^b^ |
| Deposition Index | 19.37(10.94-29.86) | 9.28(5.33-15.99) | <0.004^b^ |
| Matsuda index | 12.91(7.09-19.89) | 7.17(3.94-11.35) | <0.005^b^ |

Normally distributed data are expressed as mean ± SD, and independent samples t-test was used for comparison between groups. Non-normally distributed data are expressed as median (25th and 75th quartiles), and the Mann-Whitney test was used for comparison between groups.

^a^ : P<0.05; ^b^: P<0.01.

**Table S6. ROC curve efficacy comparison.**

| Parameter | AUC | Sensitivity | Specificity | Youden’s index | Cut-off |
| --- | --- | --- | --- | --- | --- |
| CDCA | 0.699 | 0.85 | 0.5 | 0.35 | 307.811 |
| LCA | 0.612 | 0.513 | 0.672 | 0.185 | 15.142 |
| CA | 0.578 | 0.74 | 0.437 | 0.178 | 182.0 |
| DCA | 0.544 | 0.721 | 0.383 | 0.104 | 47.0 |
| T | 0.725 | 0.644 | 0.735 | 0.38 | 1.89 |
| Combined ROC | 0.827 | 0.76 | 0.789 | 0.55 | 0.7 |

Combined ROC: CDCA、LCA combined testosterone.

**Table S7. Delong test for ROC curve efficacy comparison.**

| Predictive Indicators | Combined ROC |
| --- | --- |
| CDCA | p<0.01 |
| LCA | p<0.01 |
| T | p<0.01 |
